# From symptoms to surgery—A pathway through uncertainty and hope: An interview study of women facing ovarian surgery

**DOI:** 10.1371/journal.pone.0307666

**Published:** 2024-08-22

**Authors:** Sophia Holmlund, Elin Collins, Ann Lalos, Annika Idahl

**Affiliations:** 1 Department of Clinical Sciences, Obstetrics and Gynecology, Umeå University, Umeå, Sweden; 2 Department of Nursing, Umeå University, Umeå, Sweden; 3 Judith Lumley Centre, School of Nursing and Midwifery, La Trobe University, Victoria, Australia; University of Bremen: Universitat Bremen, GERMANY

## Abstract

**Background:**

Diagnosis of an adnexal mass might be a sign of ovarian cancer, with an overall poor prognosis. This study aimed to explore women’s experiences and perceptions of facing ovarian surgery due to an adnexal mass, and expectations on life after surgery.

**Methods:**

Individual in-depth interviews with 15 women facing ovarian surgery due to an adnexal mass. Interviews were analysed using qualitative content analysis.

**Results:**

An overarching theme, *From symptoms to surgery–a pathway through uncertainty and hope*, was identified. The theme was made up of three categories; I. The road to diagnosis, II. Striving for information and guidance, and III. Balancing emotions of hope and fear. The period between discovering the adnexal mass and surgery was often described as chaotic and difficult to manage. However, the diagnostic procedures were mostly described as timely and efficient, and participants felt safe and cared for. Person-centred care was considered crucial when being in this vulnerable situation, and the nurse navigator was described as a key person to approach for any queries. While participants expressed overall satisfaction with the information provided by health professionals, some reported a lack of information regarding the surgery’s potential impact on hormonal production and sexuality. Restrictions during the COVID-19 pandemic forced participants to attend healthcare visits alone, and some wished that health professionals had taken more responsibility for informing their relatives. Many participants focused on the positive aspects of the information gained about the adnexal mass, and that the entire situation gave perspective of what was important in life.

**Conclusions:**

Waiting for surgery on a possibly malignant adnexal mass can be very stressful, however person-centred care and the guidance of a nurse navigator can make the process more manageable. To improve women’s experience, health professionals may involve relatives more often and make sure to inform of potential hormonal loss and sexuality after ovarian surgery.

## Introduction

The time period from the first, mostly vague, symptoms through suspicion of ovarian cancer, to surgery, and a final diagnosis has been reported as a major life event where personal existence is threatened [1]. Of the gynaecological malignancies, ovarian cancer carries the worst prognosis [2] with more than 300 000 cases globally and an estimated 200 000 deaths each year [3]. In Sweden, approximately 700 women per year receive an ovarian cancer diagnosis [4]. Due to non-specific symptoms and lack of reliable screening methods [5, 6], approximately 60% of all ovarian cancers are diagnosed at a late stage (stage III and IV) [6]. Overall, less than half of all women with ovarian cancer survive beyond five years after diagnosis, although the five-year survival rate is highly dependent on the cancer histotype and clinical stage at diagnosis [6].

Because of the vague symptoms most women experience, the potential diagnosis of ovarian cancer is unexpected and a sense of confusion and surrealism has been reported [7]. The uncertainty of the diagnosis can lead to a cascade of complex reactions, for example fear for the future and of dying [8]. Although some women may feel relieved in receiving a diagnosis after a time of uncertainty, the psychological distress often escalates during the waiting time for surgery [7]. Women’s ability to prepare for the diagnosis and treatment is influenced by many factors, e.g., socio-economic conditions, personal lifestyle, partner and family relations, coping strategies, and feelings of hope. Patients have described that during the phase of preparation while waiting for surgery they have been strengthened by psychosocial support, adjusted information, and physical optimisation [1].

Women are reported to experience many difficulties during diagnostic procedures and the treatment of an adnexal mass or ovarian cancer. Lack of access to healthcare, delayed diagnosis, lack of information and knowledge, as well as uncertainty and side effects of the therapy, have been described [8, 9]. A study with ovarian cancer survivors reported that ‘systems-based challenges’ such as treatment scheduling and waiting time were experienced as the main issues within the healthcare system. Furthermore, the need to self-advocate or navigate the health system on one´s own when diagnosed with cancer was reported as time-consuming, frustrating, and exhausting [10]. Patient- or nurse navigators who are involved in each patient’s medical situation and provide logistic and emotional support, may facilitate timely access to cancer care and improve patients’ quality of life [11, 12].

Previous research has addressed a broad range of sexual concerns following gynaecological cancer diagnosis and treatment [13], such as decreased sexual activity, dyspareunia, and changes in the vagina as well as decreased libido, anxiety related to sexual performance and alterations in body image. Emotional distancing from the partner, difficulty maintaining previous sexual roles and perceived changes in the partner’s sexual interest have also been described [13]. Sexual issues in the perioperative or pre-treatment stage of ovarian cancer are rarely addressed by healthcare providers, and women often do not alert their providers of their worries and symptoms, leading to a significant impact on the quality of life [14].

### Context

In Sweden, a national program on standardised cancer care pathways (SCP) was introduced in 2015. The program aims to shorten waiting times for patients who present symptoms possibly related to cancer, reduce regional variation in management, and increase patient satisfaction and wellbeing. Each cancer diagnosis has an SCP manual including a set of alert symptoms that are meant to trigger further investigation and specify what the referral to a specialist should include. The SCP manual also consists of a maximum target number of days from the well-founded suspicion of cancer, through each step of the investigation, until the start of the first treatment [15]. Recommendations on patient support in the SCP include having a nurse navigator who will guide the patient and refer to e.g., psychosocial or existential support, dietician, or palliative care when indicated. Furthermore, effects on reproductive and sexual health should be discussed and attention should be paid to underaged children in the household [16]. Almost all women in Sweden who are currently diagnosed with ovarian cancer have been referred according to the SCP [17]. The final diagnosis of ovarian cancer is often decided after surgery and histopathological evaluation [16]. Approximately two-thirds of women referred according to the ovarian cancer SCP are diagnosed with malignancy, including ovarian, fallopian tube, abdominal-, peritoneal- or pelvic cancers as well as borderline malignancies (n≈1100 women/year) [18]. Of those, 691 women were diagnosed with ovarian, fallopian tube- and peritoneal cancer in the year 2020 [19]. The maximum target time from well-founded suspicion of ovarian cancer to the start of treatment, which is often surgery, is 24 days and 46% of women with ovarian cancer completed the diagnostic procedures within this time in Sweden in 2020 [16, 17].

Ovarian cancer surgery and prescription of medical treatment are regionally structured to six hospitals in Sweden. This study was conducted at one of these hospitals, a university hospital with a large geographic referral area. In the event of a well-founded suspicion of ovarian cancer, women are investigated according to the previously described SCP and referred to the gynaecological oncologist at the university hospital. Recommended treatment is then decided at a multidisciplinary conference (MDC) at the university hospital. The conference participants include gynae-oncologists, tumour surgeons, pathologists, radiologists, the referring gynaecologist, and the nurse navigator as well as urologists and gastrointestinal surgeons if needed. If the suspicion of ovarian cancer persists after clinical, biochemical, and radiological assessment, surgery is performed at the university hospital [16, 20]. In 2020, 206 women had ovarian surgery at the selected hospital. In 160 cases of these 206, the indication was suspected or known gynaecological malignancy and approximately two-thirds were later diagnosed with cancer.

In Sweden, patients have online access to their medical records to increase patient engagement and improve healthcare outcomes. Patients can choose to only read medical notes and laboratory results that are verified and approved by physicians or choose to read not signed medical notes with the risk of receiving medical information, including abnormal findings, before being contacted by the physician [21, 22].

### Rationale and aim

As aforementioned, there are several studies on women’s experiences after a diagnosis, as well as during and after the treatment of ovarian cancer. However, studies exploring women´s views during the phase of investigation and the pre-treatment stage of an adnexal mass are limited [1, 9, 23]. It is known that preoperative expectations are associated with postoperative quality of health [24]. Understanding the women’s experiences is essential in providing person-centred care, and the perceptions while waiting might differ from after having received a diagnosis. Furthermore, we saw a research gap in women’s views of receiving accurate information and grasping the implications of surgery, and the implication of hormonal loss and sexual functioning. The aim of this study was therefore to target the investigative and uncertain period before ovarian surgery to explore women’s experiences and perceptions, and expectations on life after surgery.

## Methods

### Study design

As the purpose of the study was to explore women’s experiences and perceptions of facing ovarian surgery, a descriptive qualitative study design was chosen. Data was derived from individual in-depth interviews. A qualitative descriptive approach has been described as a useful design to gain a rich, straight description of a particular topic where analysis stays close to the participants’ points of view [25].

### Participants

In total, 15 women who waited for ovarian surgery due to an adnexal mass were interviewed. The mean age of the participating women was 56.8 years (SD 10.9), ranging from 37 to 77 years. Seven participants were referred to the university hospital from a local hospital, the other eight were residing close to the university hospital. A majority of women included in this study had a suspected malignancy at the stage of the interview (14/15 participants), although some of them might not have been diagnosed with ovarian cancer after surgery.

### Data collection

Inclusion criteria were women waiting for surgery due to an adnexal mass at the selected university hospital. Purposive sampling was used to select and recruit participants, i.e., aiming for variation in age and place of residence. Women who did not speak Swedish were excluded due to the difficulty of capturing their experiences in an interview. Eligible women were individually approached by a surgical coordinator or a research nurse who informed the women about the study and that participation was voluntary. Potential participants were also informed that they could withdraw from the study at any time during the data collection and that participation did not affect any healthcare procedures. Participants received both oral and written information and informed written consent was obtained from all participants before the start of the interviews. All women approached for the study agreed to participate and none dropped out.

An interview guide, based on literature and clinical experience within the research group, was composed, and included questions related to the following domains: Women’s experiences and perceptions of a) seeking healthcare and receiving information from health professionals; b) the risks and benefits of surgery; c) the meaning of hormonal loss and expectations of sexual functioning after surgery; d) the risk of malignancy and how to cope with the unknown result of surgery e) quality of life and vision of the future. Before the start of data collection, the information letter and the interview guide were pilot tested with three women waiting for ovarian surgery at the selected hospital. These women were not included in the present study. The pilot study resulted in no changes to the information letter, but an additional question was added in the interview guide regarding the participants’ health information-seeking behaviour. Data collection was performed between April and December 2020. The first author, a female midwife with a PhD who has experience with the research methods in use, contacted the women who had consented to participate in the study and agreed on a place for the interview. The interviews (n = 15) were conducted by the first author, either face-to-face at the hospital (n = 7) or by phone in close connection to the surgery depending on what best suited the participants. The interviewer had no previous nor clinical relationships with the participants. After each interview, the interviewer made field notes of the content of the interview and the participant’s reactions during the narratives. The interviews were digitally recorded, and the length of the interviews varied from 19 to 55 minutes. Data saturation was assessed to have been achieved after 13–14 interviews, however, 15 interviews were finally performed as the last participants already had agreed to be included in the study. The recordings were transcribed verbatim, two interviews by the first author and the other 13 interviews by a medical secretary.

### Data analysis

The data was analysed manually using qualitative content analysis [26]. Initially, the transcribed interviews were read several times (SH and AI) to get a sense of the whole. The first author (SH) coded all the interviews, and parts of the interviews were also coded by the last author (AI). Codes were compared and agreed upon. The codes were then discussed in relation to differences and similarities, while keeping the aim of the study in mind, and sorted into sub-categories. The sub-categories were abstracted and merged into categories based on similar content. Finally, a theme was generated to link the underlying meanings of the codes, sub-categories, and categories together [26]. The preliminary analysis was reviewed by all authors and discussed until a consensus was reached.

## Results

The theme, From symptoms to surgery–a pathway through uncertainty and hope, describes women’s experience from discovering the first symptoms, their contact with healthcare, and the process of diagnostic procedures until surgery of an adnexal mass. Although the women were struggling with the uncertainty of not knowing whether the adnexal mass was malignant or not, most women reported a sense of hope and a strong fighting spirit to get well again. The theme was built on three categories and nine sub-categories presented below (Table 1). Quotes from the participants are presented together with an ID number allocated for each participant to ensure the credibility of the results.

**Table 1:** Sub-categories, categories and theme in 15 women waiting for ovarian surgery due to an adnexal mass.

Theme	From symptoms to surgery–a pathway through uncertainty and hope		
Category	The road to diagnosis	Striving for information and guidance	Balancing emotions of hope and fear
**Sub-category**	Acknowledging symptoms	Acquiring and interpreting medical information	To tell or not to tell and dealing with others’ reactions
	Enduring a whirlwind of diagnostic procedures	Appreciating person-centred care	Facing the immediate consequences of surgery
	Coping with anxiety and uncertainty	The coronavirus impact on healthcare routines	Pondering future life and sexuality

### I. The road to diagnosis

#### Acknowledging symptoms

The participants described a range of symptoms that made them seek healthcare. Lower abdominal pain, swelling of the abdomen, fatigue, sensation of heaviness in the pelvic area, vaginal bleeding and urinary problems were commonly reported. Most women described a low to medium degree of pain or discomfort, however some participants had experienced acute pain in the lower abdominal area. A few were diagnosed with an adnexal mass during a routine examination.

‘*I was quite shocked and understood immediately that—yes, I have felt for a while that there was something that wasn’t good. I have connected it to other things and thought… received signals from the body, but haven’t addressed it or else had other explanations for it.’* Participant 9

Feelings of surrealism were described when the adnexal mass was discovered, and the risk of malignancy came as a shock for most women. Despite this, some participants described that in retrospect they realised that the symptoms had been there for a while although ignored or explained by self as a common condition. Some participants also described that they were so busy with work and family life that they had not prioritised seeking healthcare.

‘*There has been a lot going on during the corona pandemic, and my mother is in a high-risk group… yes, there has been a lot of concern around that. I have my own business and a lot of work and so on, so I haven’t really thought about myself and how I was actually feeling.’* Participant 7

The gynaecological symptoms described were also reported to be typically female issues that many women endure for a long time before seeking healthcare e.g., prolapse and urinary incontinence.

‘*I realised then that I had been deceiving myself, that it wasn’t as simple as I had thought. But I hadn’t had any particular pain so I wasn’t… Really, I had been bothered by the prolapse and a bit gassy, a bit bloated, but I had never thought I had problems like that.’* Participant 11

#### Enduring a whirlwind of diagnostic procedures

Seeking healthcare was done either through the primary health centre, the gynaecological outpatient clinic at the hospital, the emergency department, or a private gynaecologist. Most participants described that the contact with healthcare led to a timely examination by a doctor and that a cascade of examinations was initiated at the discovery of the adnexal mass.

*It was great. She did an ultrasound and she saw that there was a large cyst… she took blood samples and enrolled me on this fast track… what’s it called, you know… standardised cancer care pathway.’* Participant 1

A few participants who were seeking healthcare at the primary health centre or the emergency department felt that their symptoms were not taken seriously and that they had to struggle to get a medical appointment within a reasonable timeframe. Perceived gender aspects of health seeking behaviour and receiving a doctor’s appointment were also described.

*It can be down to women themselves that you haven’t… you don’t demand in the same way. Well, if a man decides to go to the doctor… I don’t know, but it feels like they get help faster, get paid more attention.’* Participant 4

A majority of women perceived the diagnostic procedures as timely and efficient after being referred to the gynaecological clinic, and they felt safe and cared for during this process.

*‘I’ve felt that, oh, I’m well taken care of… and it’s nice because I like to be in control… I usually fix things. I arrange, fix, and take care of the logistics and all this, but now I can’t do that. I just have to rely on others to do this and know this. I get to go along safely and it’s going great.’* Participant 15

Many participants described that they were surprised by the short time between the diagnosis of the adnexal mass to the appointment for surgery. The nurse navigator was described as a central person for the participants during the diagnostic procedures. Participants reported that it was very positive that the nurse navigator organised all the medical investigations and tests, and that they knew who to contact if they had any questions.

*‘Really, I’ve had great help from the nurse navigator who has pulled me through all of this and made everything happen incredibly fast…’* Participant 8

#### Coping with anxiety and uncertainty

Many participants described feelings of shock and surrealism when informed about the adnexal mass, especially when they did not feel sick. Although some reported that it was hard to understand the severity of the diagnosis, some also perceived that feeling healthy and full of energy was a good sign and conferred a lower risk for the adnexal mass to be malignant. A coping strategy described by some participants was to see the positive aspects and not to think of cancer until the final diagnosis was given. A few participants described not being worried at all. However, many participants struggled with the uncertainty of the results after surgery.

*‘It’s never nice to wait… but it is… there is no point in looking into and taking things in advance until you have the answer… It’s like that, you shouldn’t paint the devil on the wall.’* Participant 5

‘*Really*, *I was so shocked and sad… it wasn’t fathomable and the whole afternoon and evening was just… It can’t be explained*, *it was just a lot of panic attacks and thoughts… for the kids and your life altogether… it was just chaos*.*’* Participant 8

The time period from the discovery of the adnexal mass to surgery was often described as chaotic and very hard to manage. Some tried to distract themselves by working and keeping up with their normal routines. Others described that they immediately paused their work and decided to focus on being with their family and prioritised what they felt was most important in life. Because of the number of examinations and tests, some also expressed difficulties being at their workplace when sometimes having to attend examinations and tests with short notice. The process of diagnostic procedures was described as very stressful, despite this the vast majority reported that they understood that the examinations and tests were required and that being continuously informed about the process and results made it easier to cope.

### II. Striving for information and guidance

#### Acquiring and interpreting medical information

Most participants were satisfied with the information given by health professionals throughout the whole journey of diagnostic procedures. However, a few participants had experienced that the information about the adnexal mass and a suspected malignancy was given in a non-optimal situation, for example after a long waiting time at the emergency department or without any family member or support person present. Some had received both oral and written information about the surgery although solely oral information was most common. A few participants described that they lacked understanding of what organs were going to be removed during surgery, and that written information could have helped them to understand, especially as they were in shock when the information was given. It was also understood that the doctors could not give complete information since they neither knew the final outcome of the surgery. However, participants appreciated the doctor’s honesty regarding their knowledge of the situation, and that they were informed about all the steps in the diagnostic procedure.

*‘It has been very quick, very quick how the information has been conveyed back to me. I’ve received information over the phone and then at the gynaecology department by the doctor, and have been given information all along so…. I think it has worked very well.’* Participant 9

The need to search for additional information through the Internet differed between the participants. Some described a need to read about other women’s experience of having an adnexal mass with suspected malignancy, and gaining additional information through the Internet was a way of coping with the situation. Others described a conscious choice of not searching for additional information on the Internet as this could add even more stress to the situation.

‘*I chose not to use Google. I haven’t read anything and it’s also a protective mechanism… If they say that this is malignant then I can find out what I can do. I don’t want to become upset before I have all the facts.’* Participant 2

Several participants also expressed that they had accessed their medical records online, and that reading their medical records helped them to confirm their understanding of the information given orally by the doctor. Reading the medical records was also a way of keeping track of what happened during the diagnostic procedure; however, it was also understood that reading medical text with a lack of understanding of what it meant could make things worse.

‘*I want to know facts, I want to… I’m curious so I want to try to understand… so, it might not be the smartest, it might be better if a doctor explains but it is also my own choice to go* [to the online journal] *and read what isn’t signed or attested, it’s my own choice.’* Participant 11

#### Appreciating person-centred care

Many participants described that they fully trusted the health professionals and felt that they were treated in an empathic and professional way. For the participants who had sought healthcare at a local hospital, most described the referral process to the university hospital as smooth and they felt that there was good communication between the two hospitals. A few participants reported a contradictory experience with a lack of information between different healthcare departments and a sense of not being involved in the healthcare process. One participant described that the health professionals focused on the organisation instead of the individual patient and that there was a lack of person-centred care. Another participant described that the surgery was cancelled with short notice because of a more urgent case, something that was fully understood by the participant although putting her own life and planning on hold.

‘I don’t know how many doctors have been involved, but I haven’t been in focus, the healthcare has been the focus… it’s a d**n difference…the machinery called healthcare was what was important; that referral and those labs and so on…’ Participant 2

Continuity of care was described as important, as having different doctors could increase the risk of misunderstanding and confusion. In these situations, a named nurse navigator mediated the information needed, and was there as a known person to turn to. It was also explained how the health professionals’ mode and behaviour affected their wellbeing.

‘*I thought the doctor was so positive when she rang, this gave me so much strength.’* Participant 14

‘*I know… healthcare goes on… but I felt that the human contact was handled really*, *really well*.*’* Participant 15

Some participants had been informed or asked if they wanted to speak to a counsellor, however, the majority felt that it was not needed until they had the final diagnosis. Talking to the family was perceived as enough at this stage. Despite this, some participants expressed that it was important to be informed that the counsellor was available, as some women may need it during the diagnostic procedures.

‘*They have said that I can call as much as I want and there is a counsellor, but I feel… counsellor… I have nothing I need to talk about right now. It is what it is, and I don’t want to go too deep into what it could be, then I’ll just get more worried.’* Participant 7

#### The coronavirus impact on healthcare routines

The COVID-19 pandemic influenced the participants’ experience of waiting for surgery in different aspects. Most participants described that the restrictions due to the pandemic forced them to attend all healthcare visits by themselves, without a support person. Receiving medical information on their own was experienced as challenging and participants described difficulty in conveying the information to their relatives, especially when they felt shocked. It was also reported that this situation made it extra challenging for their relatives, as they were not able to ask their questions to the health professionals. Some expressed that it would be good if the health professionals had offered to contact their next of kin to provide information. Despite feelings of loneliness during the healthcare visits, all participants were very understanding of the restrictions that were put in place due to the pandemic.

‘*I think it is appropriate that we work together to minimise the number of healthcare visits. Those who don’t need to be there shouldn’t be there and I can’t blame healthcare for that…’* Participant 11

A few participants described that they had postponed the initial contact with healthcare due to the risk of becoming infected with the coronavirus. Not being able to meet family and friends in this challenging time of life was putting additional stress on the situation. It was also reported how some participants tried to isolate themselves the time before the surgery, to avoid being infected. The risk of the surgery being postponed due to the extra pressure put on healthcare during the pandemic was also described as demanding for some participants.

‘*It feels tough, the whole process with corona and having to go alone all the time and hearing it all… There is so much information so when you try to retell it it’s like…oh, I can’t remember what they said. So, I’ve felt that I’ve been alone in the process.’* Participant 14

### III. Balancing emotions of hope and fear

#### To tell or not to tell and dealing with others’ reactions

Differing attitudes towards talking about the adnexal mass to family, friends and colleagues were described. Some reported that they had only talked about the severity of the situation to their partner and that it was too early to talk to others about it, as there was no final diagnosis yet.

*‘I haven’t told anybody but the closest. I don’t want any questions since I don’t know… know anything yet and it’s just hard to have people… How are you? Have you heard anything yet? I don’t want those questions now… It’s enough just focusing and staying above the surface. Should I have to keep a facade… well I don’t have the energy. Then it is better to lock the door and be alone.’* Participant 7

Participants having a partner relationship also described their partner’s difficult role of trying to be both supportive and at the same time worried and sad. A few also expressed that they felt that their partner was holding back their own emotions and fears to not be an additional burden for the woman. Most participants reported that it was natural to tell their closest family and friends about the situation. How and when to tell their children about the surgery of the adnexal mass was sometimes described as very challenging. Although most participants had adult children, some reported that it was a balancing act of providing clear information and not causing too much worry. A few participants described that they were holding back with the information that there was a risk of malignancy, to protect their children. Others described that they had decided to be honest and open with their children, expressing that hiding information could only make things worse.

‘*I was shocked… and I think what you worry about the most is how, but how do I tell the children? … of course, I had to tell my husband. We have adult children so I chose not to tell them, I still haven’t told them about that. I have told them about surgery though.’*

Participant 4

For those with younger children, it was expressed that it was very important to communicate with the children about the illness so they could understand why their mother was sad. One participant also described how she had contacted the children’s teachers to inform them about the illness, if the children would behave differently at school.

Some felt that it was too hard to deal with the reactions and questions of others in this early stage of diagnostic procedures. Others meant that it is impossible to hide an illness, and talking about the situation with family and friends gave them support to cope with the situation. One participant had decided to write about her illness on social media to reduce the risk of spreading false rumours, something which also gave her support of others who had experienced similar situations.

‘*I wrote about it on Facebook, because I know there have been a lot of rumours going around since I haven’t been to work. There have been rumours that I’m pregnant… so I thought I might as well be open and honest.’* Participant 10

#### Facing the immediate consequences of surgery

Although some participants expressed fear of the surgery and feelings of lack of control with undergoing anaesthesia, the majority of participants described that they wanted to have the surgery done as soon as possible to get rid of the adnexal mass. Having the surgery was described as the first step to getting well again, and this could decrease the risk of having other problems with the reproductive organs in the future.

‘*I’m thinking a bit more… the relief of getting this cyst out is step number one. I have to* [have the surgery]*, no matter what answers I’ll get. It might be bad but I still have to get it out* [the cyst] *for this to turn around.’* Participant 9

Removing the reproductive organs was mostly reported without major worries for participants who had already passed their reproductive period in life. Many participants expressed that they did not need the uterus and the ovaries anymore and that these organs did not mean more to them than other organs in the body. However, most participants pointed out that this situation would have been totally different had they been younger and not already had children. These participants also perceived that femininity had nothing to do with the reproductive organs. In contradiction, a few participants described a strong emotional connection with the reproductive organs. Despite not needing the organs for reproduction anymore, losing them was described as being mutilated as a woman.

*‘Really it is something you are quite moved by, you are mutilated as a woman in a way. It can’t be seen but I know… the knowledge that it is something of what makes me a woman that I’m losing. It is a physical loss, I really think it is.’* Participant 3

#### Pondering future life and sexuality

The majority of participants had a bright vision of their future and were trying to focus on the positive aspects of the information they had gained about the adnexal mass. It was also reported that this situation gave a perspective of what was important in life. Some participants had already thought of making new decisions for their future, for example, changing workplace and prioritising themselves more. Fear of what the future could bring was also described and some participants reported that they did not want to think about the future.

‘*I can’t wait for the day when you can look back* [at this situation]*… The challenge… which might sink you, might also strengthen you. To re-evaluate and appreciate things differently.’* Participant 10

Many participants felt uncertain about whether their hormonal production would be affected, and if so, how a lack of hormones could affect them after having the ovaries removed. Although it was commonly described that loss of hormones was secondary to surgery and the risk for malignancy, many participants reported a lack of information on how the surgery could affect hormonal loss. The lack of information by health professionals about the hormonal consequences of the surgery was sometimes perceived as a sign that this was not an area of concern. Some participants also reported that they relied on healthcare to solve this, if the lack of hormones would cause them any problems after surgery. Fear of estrogen therapy concerning the risk of malignancy was also raised by a few participants.

The impact of the surgery on sexuality was reported to only rarely be brought up by health professionals. A few participants had received written information about the consequences on sexual health and some had asked the doctor about sexual issues in relation to the surgery. While some participants expressed that they had not thought about the consequences on sexual health at all, some described that this was an area of concern.

*‘I still want to be able to feel lust… and it hasn’t been discussed, and I’ve forgotten to ask about it. But I’ve assumed it is not where it sits* [the organ which is going to be removed].*’* Participant 6

## Discussion

This study explored women’s experiences and perceptions of the investigative and uncertain period before ovarian surgery due to an adnexal mass, and expectations on life after surgery. The theme *From symptoms to surgery–a pathway through uncertainty and hope*, describes women’s experiences and perceptions during the process from experiencing the first symptoms, their contact with healthcare, and the process of diagnostic procedures until the ovarian surgery. The women commonly described a feeling of surrealism when the adnexal mass was discovered and when they were informed about the risk of malignancy. It is well known that the symptoms of ovarian cancer are vague and mostly unspecific. In a study examining the awareness of symptoms and risk factors of ovarian cancer, more than half of a sample of 857 women were unfamiliar with the potential warning signs. However, the authors conclude that by teaching and encouraging healthcare providers about the symptoms and by raising women’s awareness, ovarian cancer may be detected at an earlier stage [27]. Some participants in our study reported that they endured the symptoms for a long time before they contacted healthcare as they thought that these were female issues that women had to live with, or they were too busy to seek healthcare. This is in line with the results from a Danish study where more than 26,000 women completed a questionnaire regarding barriers to contacting a general practitioner (GP) when experiencing gynaecological cancer symptoms, for example, pelvic pain and bleeding during intercourse. The authors found that less than 35% of women who reported gynaecological cancer alarm symptoms contacted a GP, and being too busy was the most prevalent reported barrier. Low age and low socioeconomic status were associated with higher odds of reporting barriers to seeking healthcare [28]. Being too busy to seek healthcare may also be explained by denial of symptoms and a strategy for protection against stressful feelings [29].

Despite that less than half of all women in Sweden have completed the diagnostic procedures within the maximum target time set in the standardised cancer care pathway (SCP), [17] participants in our study were generally satisfied with the diagnostic procedure and experienced it as timely and efficient. An Australian study, exploring women’s experience of healthcare services and preferences during the diagnostic phase of ovarian cancer, concludes that prompt and responsive healthcare services that provide patient-centred information, employ relational communication and prepare women for survivorship could improve women’s experiences [7]. Patients’ satisfaction with healthcare after being investigated through a SCP in Sweden is explored in a national survey annually. The results from 2020–2021 show that patients were generally satisfied with the healthcare during the diagnostic procedure, especially those who received a cancer diagnosis. Patients also experienced increased accessibility to healthcare during the COVID-19 pandemic and reported high levels of satisfaction with the support received by healthcare. However, support to their family members was reported at a reduced rate compared to the years before the pandemic, according to the SCP follow-up [30]. Insufficient involvement and support of family members or other support persons were also described by participants in our study. This result is affected by the restrictions that were put in place worldwide due to the pandemic, including restrictions on accompanying support persons during hospital and outpatient clinic appointments. The combination of being investigated for a possible gynaecological cancer and the fear and the restrictions due to the coronavirus has been described as ‘managing a dual challenge’, resulting in feelings of loneliness and vulnerability [31]. Participants in our study reported an explicit understanding of the restrictions that were put in place due to the pandemic. Despite this, ideas of how health professionals could have been more active in contacting and giving information to their next of kin, with their consent, were brought up. Some reported that the shock of receiving information about a suspected malignancy affected their ability to remember the information that was given, which led to difficulties in explaining the situation to their family members. Participants were also worried that their family members lacked someone to ask about the illness and feared that their family members did not want to place a burden by asking the one who was ill. Holt et al. [32] interviewed 16 women and 16 relatives at different times during the prediagnostic period of gynaecological cancer. They found that both women and relatives need involvement, an overview of the treatment process as well as readily accessible information when needed. Relatives also need someone to talk to and advice on how to communicate about cancer if there are children in the family [32]. In Sweden, it is the healthcare’s responsibility to make sure that children with a sick parent are informed about the situation and that advice and support are based on the child’s individual needs [33, 34]. A few of the participants in our study had younger children. However, participants with adult children were also worried about if, or how, they should give information about the risk of cancer. In line with the findings of Holt et al. [32] some of the participants wished to talk with their relatives about the diagnostic process and their worries, while others reasoned that they first needed to verify the suspicion and the severity of the illness.

The nurse navigator was described as having an important role in organising the medical investigations and tests, and a known person to turn to in case of questions. Other studies have also reported the value of having an assigned nurse navigator during the diagnostic phase of cancer [35] as well as during treatment, with higher patient satisfaction and enhanced experience [36]. In addition, having a nurse navigator who works in a multidisciplinary team is also associated with reduced waiting time between abnormal screening results to the first treatment [35]. The recommendation of a nurse navigator in the Swedish SCP can therefore be considered a valuable healthcare intervention [37].

When discussing the significance of removing the reproductive organs, most participants in our study did not associate it with losing their femininity, although a few explained that they had a strong emotional connection with their reproductive organs. In accordance with our results, another study from the Swedish setting found that some women considered the uterus as a vital part of being a woman. Other women felt that removing the uterus could be done without grief when the function of it was not needed anymore [38]. In a study from Brazil exploring women’s experiences of coping with the changes imposed by gynaecologic surgery, women came to reassess the perception of themselves and their social environment and felt mutilated after surgery [39]. Losing reproductive organs can also be closely related to aspects of sexual health. According to The World Health Organization sexual health is: ‘…a state of physical, emotional, mental and social well-being in relation to sexuality; it is not merely the absence of disease, dysfunction or infirmity.’[40]. Participants reported that the impact of the surgery on sexuality was seldom brought up by health professionals and lack of information was sometimes perceived as a sign that this was not an area of concern. Sexual difficulties and poorer quality of life are, however, commonly described among women with ovarian cancer [14, 41, 42]. Although the participants in our study were not diagnosed with ovarian cancer at the stage of the interview, they were all going through ovarian surgery which could, depending on the extent of the surgery, age and the outcome of the diagnosis, greatly affect their sexual health. There is a need for women to get proper information about the potential impact of ovarian surgery on sexuality, including those women who are not currently in a partner relationship. However, providing relevant information can be described as a balancing act for health professionals. In a Swedish study exploring patients’ views of the content of information letters received before benign gynaecological surgery, patients found it very difficult to assimilate the most important information due to information overload [43].

A limitation of this study is that data collection was only performed at one hospital, although the participants resided in a greater geographic area. Due to the implementation of the Standardised cancer care pathways in Sweden, it is, nevertheless, likely that women at other hospitals have received similar care. Limitations that may affect transferability to other populations include the lack of sociodemographic information on the participants, e.g., education, civil status and country of birth. Women who did not speak Swedish fluently were excluded from participating in this study affecting transferability to the wider population. It is plausible that non-Swedish-speaking women would have had other experiences and needs in terms of information during the diagnostic procedure. There is also a risk of selection bias as the surgical coordinator or the research nurse who recruited women to the study might have chosen to ask only women they thought were suitable to tell their story, e.g., younger women and women who seemed to be in a psychologically stable state even though the intention was to include all.

Data collection was performed during several months of the COVID-19 pandemic, and restrictions in healthcare might have changed during this period which may have affected the results.

### Conclusion

Facing ovarian surgery due to an adnexal mass was perceived as surrealistic and very challenging with feelings of shock about the risk of malignancy. Most women experienced the diagnostic procedure as timely and efficient, with the nurse navigator as a key person, and they felt safe and cared for during this process. The study results emphasize the importance of person-centred information and care in the time period from the discovery of an adnexal mass until surgery, and that relatives need to be informed and involved in this process, if the woman so wishes. Special attention should be given to women who have children. Proper information about potential hormonal loss and sexuality in relation to ovarian surgery is required, and health professionals should take the initiative in this discussion to ease the burden for the women.

## List of abbreviations

GP: General Practitioner
MDC: Multidisciplinary conference
SCP: Standardised cancer care pathways
